# Insights into the influence of diet and genetics on feed efficiency and meat production in sheep

**DOI:** 10.1111/age.13383

**Published:** 2023-12-19

**Authors:** Steffimol Rose Chacko Kaitholil, Mark H. Mooney, Aurélie Aubry, Faisal Rezwan, Masoud Shirali

**Affiliations:** ^1^ Institute for Global Food Security, School of Biological Sciences Queen's University Belfast Belfast UK; ^2^ Agri‐Food and Biosciences Institute Hillsborough UK; ^3^ Department of Computer Science Aberystwyth University Aberystwyth UK

**Keywords:** carcass quality, diet, feed efficiency, genetics, heritability, meat quality, sheep

## Abstract

Feed costs and carcass yields affect the profitability and sustainability of sheep production. Therefore, it is crucial to select animals with a higher feed efficiency and high‐quality meat production. This study focuses on the impact of dietary and genetic factors on production traits such as feed efficiency, carcass quality, and meat quality. Diets promote optimal sheep growth and development and provide sufficient protein can lead to higher‐quality meat. However, establishing an optimized production system requires careful consideration and balance of dietary parameters. This includes ensuring adequate protein intake and feeding diets with higher intestinal absorption rates to enhance nutrient absorption in the gut. The study identifies specific genes, such as *Callipyge*, *Calpastatin*, and *Myostatin*, and the presence of causal mutations in these genes, as factors influencing animal growth rates, feed efficiency, and meat fatty acid profiles. Additionally, variants of other reported genes, including *PIGY*, *UCP1*, *MEF2B*, *TNNC2*, *FABP4*, *SCD*, *FASN*, *ADCY8*, *ME1*, *CA1*, *GLIS1*, *IL1RAPL1*, *SOX5*, *SOX6*, and *IGF1*, show potential as markers for sheep selection. A meta‐analysis of reported heritability estimates reveals that residual feed intake (0.27 ± 0.07), hot carcass weight (0.26 ± 0.05), dressing percentage (0.23 ± 0.05), and intramuscular fat content (0.45 ± 0.04) are moderately to highly heritable traits. This suggests that these traits are less influenced by environmental factors and could be improved through genetic selection. Additionally, positive genetic correlations exist between body weight and hot carcass weight (0.91 ± 0.06), dressing percentage (0.35 ± 0.15), and shear force (0.27 ± 0.24), indicating that selecting for higher body weight could lead to favorable changes in carcass quality, and meat quality.

## INTRODUCTION

The sheep farming industry is characterized by a wide range of farming and feeding systems that cater to the adaptability of these animals. Farmers can choose from extensive grazing diets to highly intensive total mixed rations, depending on their individual needs. Due to the range of breeds and crosses, sheep are raised in various geographical regions, making them suitable for any environment. Their high nutritional value and environmental adaptability make them an excellent choice for farmers looking to raise healthy and productive livestock (Cannas et al., 2019). However, the cost of sheep meat production has increased as feed costs account for up to 65%–70% of overall production expenditure (Zhang et al., 2017) This has resulted in many farmers quitting farming, resizing their flocks, or changing to dual‐purpose breeds. In recent years, there has been an increase in interest among sheep meat producers in breeding ewes and lambs that can maintain production levels while eating less feed; this capacity is referred to as feed efficiency (FE) (Meyer et al., 2015). It has been proven that selecting animals with higher FE can result in lower feed costs without compromising the performance of the animal (Ellison et al., 2022; Paula et al., 2013). Improved FE can also reduce the environmental effects of production systems due to the lower feed consumption, decreased excretion of manure, and reduction in methane emissions (Dumont et al., 2018). Breeding programs that incorporate genetic selection of feed‐efficient animals are another sustainable technique for increasing productivity whilst lowering feed intakes (Do & Haja, 2018). In addition to efficient feed utilization, sheep production systems are also concerned about end product quality, particularly of the carcass and meat, which includes several traits such as dressing percentage, carcass conformation, fatness, meat tenderness, color, flavor, and water holding capacity (Prache et al., 2022).

There are many components that influence FE, carcass quality, and meat quality, out of which diet and genetics are recognized as two significant elements. Ellison et al. (2022) observed that the type of diet fed to sheep had influence on the feed intake and average daily gain (ADG) and forage‐based pelleted diets were associated with greater feed intake. Additionally, when sheep are fed well, they tend to accumulate more adipose tissue, which is lower in water content and higher in lipid content (Murphy et al., 1994). In case of genetic factors, several genes and quantitative trait loci (QTL) have been reported to influence the FE and production traits with one of the genes, calpastatin (*CAST*) showed strong relationships with many factors in Awassi sheep, including growth, carcass quality, and meat quality (Jawasreh et al., 2017).

The objective of this review was to provide a comprehensive summary of the existing data from studies conducted on the impact of dietary and genetic factors on FE and carcass and meat quality traits in sheep. Using a systematic approach to the identification and review of published studies in this field, a key aim was to establish a clear and concise overview of the current state of knowledge in this field and to highlight the key findings that have emerged from recent research. By synthesizing this information and through performance of a meta‐analysis of reported heritability of important traits, we hope to provide valuable insights into the factors that can affect the production of feed‐efficient sheep with high‐quality carcass and meat, that can inform future research and management practices in this area.

## MATERIALS AND METHODS

### Literature search strategy

**FIGURE 1:**
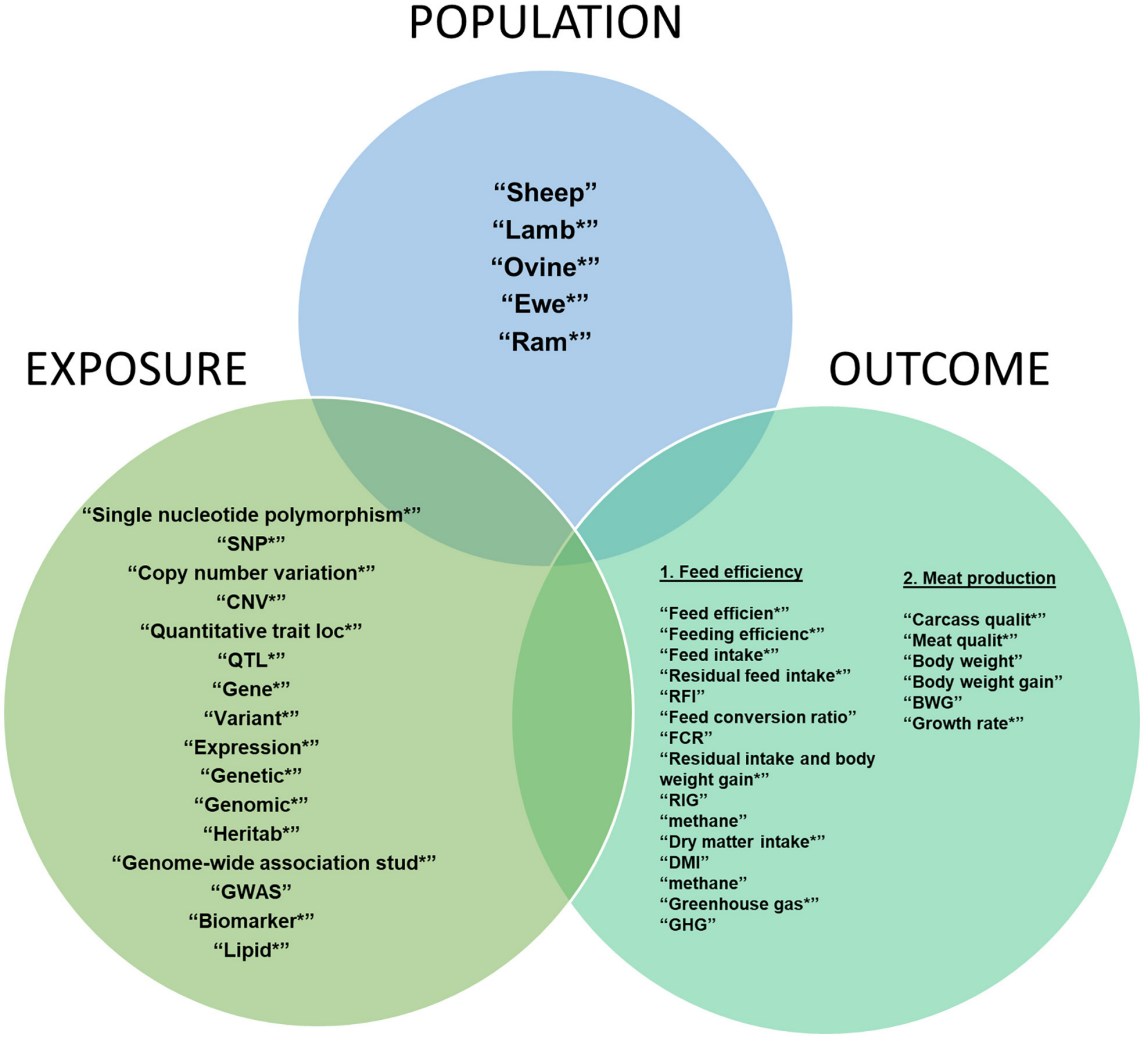
Population, exposure and outcome framework used in the literature search to identify systematic reviews on sheep feed efficiency, carcass quality, and meat quality traits.

The search strategy was based on the preferred reporting items for the systematic reviews and meta‐analyses (PRISMA) framework (Moher et al., 2010). Firstly, the research question—to understand how the type of diet fed to the sheep influence the economically important traits such as FE, carcass quality, and meat quality in addition to the genes that influence these traits were decided, and available literature were then searched to generate the population, exposure, and outcome components of the research question (Figure 1). The population was defined as ‘Ovine’ with an exposure of ‘Genetics’ and the outcome ‘feed efficiency‘, ‘carcass quality‘, and ‘meat quality’ traits. A combination of four different databases were used for the search strategy namely EBSCO, PubMed, Scopus, and Web of Science (accessed on 13 September 2023). The database settings were adjusted to improve search performance and drop irrelevant results. Only journal articles from the years 1994–2023 were retrieved from the databases.

### Screening of studies

**FIGURE 2:**
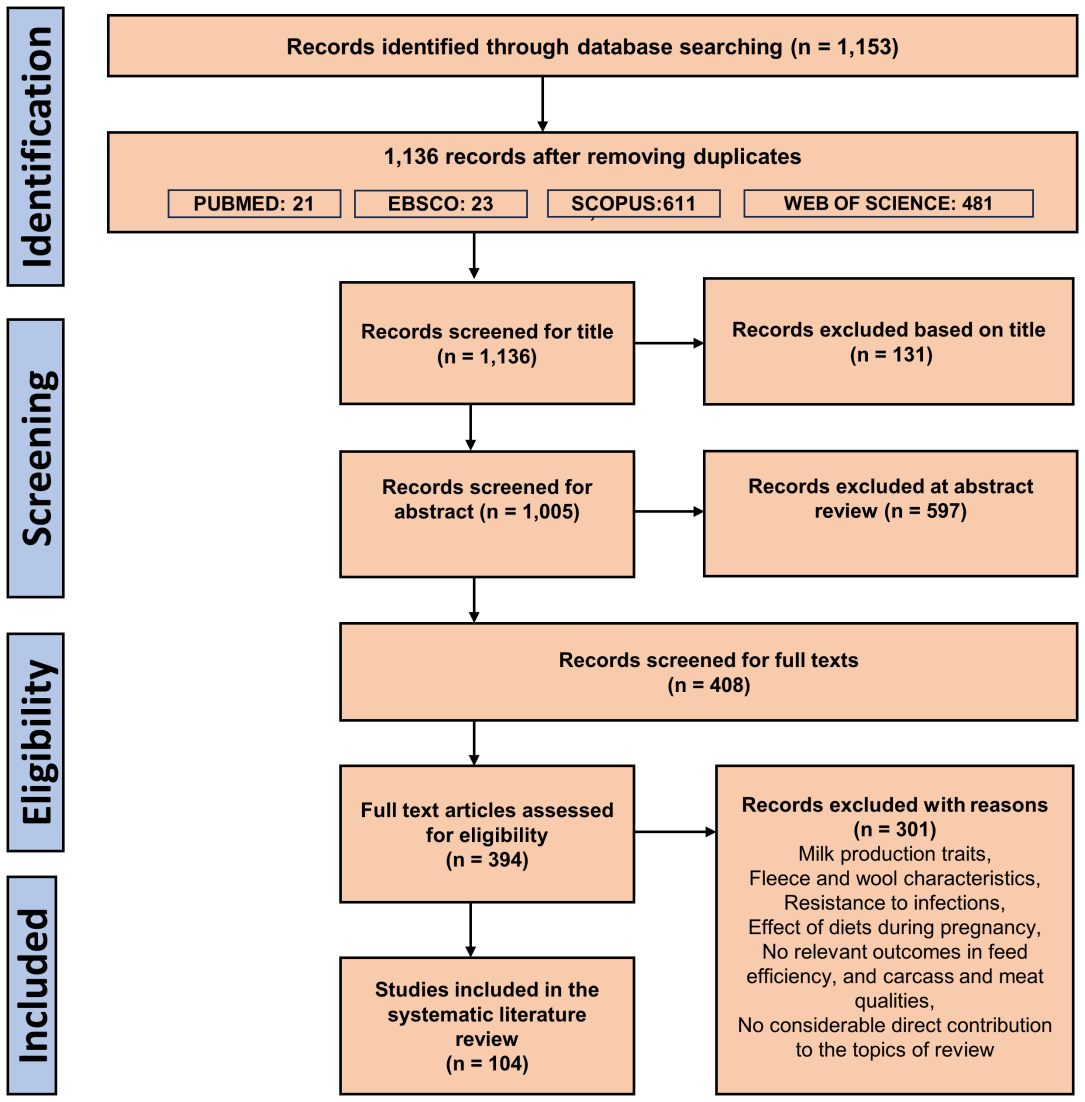
PRISMA diagram: this diagram shows how studies are included in reviewing the scope of systematic reviews in the literature on sheep feed efficiency, carcass quality, and meat quality. The number of studies chosen for screening and number of studies retained and removed after screening are displayed.

The following criteria were used to find the studies that were eligible for the review: (1) primary research; (2) articles written in English language; (3) studies involving sheep, lambs, ewes, or rams; and (4) studies describing genetics or genome‐wide association studies or genomic technologies. Two researchers independently evaluated all qualifying studies to establish suitability for full review by manually checking database search results and analyzing the titles and abstracts. A total of 1153 studies were initially returned by the search strategy (Figure 2) from which duplicates were removed. Additionally, studies were removed if the subject title was not ‘ovine, sheep, rams, ewes or lambs’ related. Upon completion of the initial screening process, a thorough examination of both the title and abstract sections of relevant literature were carried out. To ensure a comprehensive review, a rigorous exclusion criterion for the literature review was implemented. Specifically, studies that addressed one or more of the following key factors: FE, carcass quality, or meat quality were considered. Consequently, any studies that focused on topics outside of these parameters, such as the historical context of sheep farming and production, or traits unrelated to the scope of this review, such as milk production and wool/fleece characteristics, were excluded from consideration. This screening approach enabled us to provide a thorough and focused review of the relevant literature and guaranteed that the conclusions drawn were highly relevant to the main dietary and genetic factors under investigation. Certain studies were excluded from the review due to their inability to identify notable impacts of various diets on factors such as FE, carcass quality, and meat quality. Specifically, diets that did not yield significant changes in growth performance or meat fatty acid profile were not included in the review. Consequently, it was challenging to arrive at conclusive findings regarding the effects of diets on these factors. After careful consideration, 104 studies were selected for review, which provided a comprehensive evaluation of the several factors that affect FE and other quantitative traits, including dietary and genetic influences. The full texts of the chosen studies were thoroughly reviewed to confirm that they aligned with the scope of the review.

### Categorization of studies

Within the spectrum of identified studies, dietary (*n* = 47), genetic parameters such as heritability and correlations (*n* = 8) and genes reported to influence sheep FE and productivity traits (carcass and meat quality; *n* = 31) were clearly identified as key thematic aspects of interest, with an additional 18 studies focused on biological markers including genomic, proteomic, and metabolite markers associated with the traits. Although the search strategy did not specifically include proteomics and metabolomics, studies related to these technologies were still retained due to their potential value in studies based on FE and production traits. The scarcity of research utilizing these technologies makes it imperative to incorporate them into the review to provide comprehensive information to fellow sheep researchers about their potential in breeding programs. By doing so, it can contribute to the advancement of sheep breeding and ensure the production of high‐quality livestock.

### Meta‐analysis of heritability estimates

A meta‐analysis was performed on heritability estimates of sheep FE, carcass quality, and meat quality traits using the R package meta (Borenstein et al., 2021). Forest plots were generated for each of the traits to visualize the results. Each square in the forest plot represents an individual study with the size of the square indicating weight given to that study. The sample size of study determines the weight with larger square indicating greater impacts on the mean effect size. The horizontal dashed line in the plot shows the study's 95% confidence interval.

## RESULTS AND DISCUSSION

### Dietary factors influencing sheep FE, carcass quality, and meat quality

There has been a considerable number of studies (Table 1) conducted to assess the impact of various diets on FE, fatty acid composition, and lipid profile of sheep carcasses, as well as the quality of the resulting meat. Proper nutrition plays a pivotal role in the growth and development of lambs (Sayed, 2009). The overall well‐being, health, and future productivity were all significantly impacted by diet quality and quantity as observed in Table 1. As such, it is crucial to ensure that lambs receive the appropriate nutrients and supplements necessary for their optimal growth and development. Carcass and meat quality are also heavily impacted by diet type with the proportion of adipose tissue, bone, and muscles, which are growth indicators, all having an impact on the resulting carcass weight (Silva et al., 2016). Besides, there has been an increasing interest among consumers to gain an in‐depth understanding of the nutrient composition of meat and its impact on human health, in addition to its physicochemical and sensory qualities. The biochemical makeup of meat, which encompasses amino acids, vitamins, lipids, fatty acids, and other constituents, plays a vital role in determining its overall nutritional value (Kowalczyk et al., 2022). Whilst meat is thought to have harmful impacts on human health because of its high saturated fatty acid (SFA) content, unsaturated fatty acids (UFA) have been shown to take part in several metabolic activities advantageous to human health (Ladeira et al., 2012).

**TABLE 1:** Growth performance, carcass quality, and meat quality of sheep fed different diets.

Type of diet	Feed efficiency/growth performance	Lipid composition or fatty acid profile	Carcass qualities	Meat qualities	Reference
Vitamin A (6600 IU/kg of diet)	No effects on feed intake, ADG and body weight of the animal	Increase in total intramuscular lipids (25% more for high vitamin A fed lambs), and oleic acid concentrations. Decrease in linoleic acid concentrations	Greater marbling scores (514) and more extractable intramuscular lipids in carcass	‐	Arnett et al. (2007)
Corn straw and finger millet straw	Improved body weight (42.04 ± 2.88 for corn straw and 42.26 ± 5.22 for finger millet straw) and ADG (192.21 ± 22.27 for corn straw, 193.46 ± 34.48 for finger millet straw)	‐	No significant effects	‐	Chen et al. (2020)
Various levels of lycopene from tomato extracts (50, 100, 200 mg lycopene/kg)	Greater dry matter intake (989, 1055, 1023 g/day) and ADG (9%, 12%, 7%)	Increased PUFA with 100 mg lycopene/kg (6.25), decreased SFA with 100 mg lycopene/kg (53.86)	Higher hot carcass weight (19.28 kg), no significant difference in carcass marbling ratio and loin eye area	Improved oxidation stability of meat	Jiang et al. (2015)
Various levels of licuri cake g/kg dry matter (low (Knapik et al., 2017) medium (Zhang et al., 2017), and high (260))	Improved final body weight (35.0 kg) with elevated levels, total weight gain (13.3 kg) with medium levels, ADG (146 g/day) with medium levels and feed conversion (9.08 g/g) with elevated levels	‐	No significant effects	No differences in cooking loss, and shear force, but meat color index was increased (L* = 36.8, a* = 24.4, b* = 11.4) with elevated levels	Bagaldo et al. (2019)
Dehydrated lucerne hay and 8% soybean oil	Decreased feed intake, ADG (184 g) reduced growth rate. Increased FCR with lucerne and soybean oil (7.20)	Proportions of all *trans*‐isomers, *cis*‐12, LA, n‐6/n‐3 fatty acids increased, supplementation of oil decreased *cis*‐9 DPA	Supplementing oil resulted in decrease of muscle proportion in carcass and increase in kidney knob channel fat.	No significant effects	Santos‐Silva et al. (2004)
Crude protein and energy concentration of diets based on barley and canola meal with and without fish meal	No significant effects observed	‐	Increased carcass weight (2.2 kg heavier carcass) with high energy diet but not dressing percentage	‐	Beauchemin et al. (1995)
Lipid source (grass pellets, cereal based concentrate, cereal based concentrate enriched with oilseed rape, cereal based concentrate enriched with fish oil)	Highest dry matter intake with grass pellet (896 g/kg), lowest in cereal‐based concentrate enriched with fish oil (835 g/kg)	‐	High carcass weight gain (g/kg DMI) with cereal based concentrate, lower carcass weight gain with cereal based concentrate enriched with fish oil (129 g/kg DMI), and grass pellets (73 g/kg DMI)	‐	Annett et al. (2011)
Selenium‐yeast and chromium‐yeast (g DM/day)	No significant differences in feed intake, ADG with selenium‐yeast, decrease in feed intake with chromium‐yeast (mean around 1212 g DM/day)	‐	No differences with selenium‐yeast, Linear increase in protein content in the carcass and fat free carcass with increase in chromium‐yeast	‐	Domínguez‐Vara et al. (2009)
Spineless cactus (0, 150, 300, and 450 g/kg)	Increase in ADG with 300 and 450 g/kg (267 and 265 g/kg ADG)	Increase in oleic fatty acids and total MUFA. Linear increase in MUFA/SFA	Increased hot carcass yield (49.3%), cold carcass yield (48.3%) with 450 g/kg spineless cactus and carcass compactness index (0.26 kg/cm) increased with 300 g/kg spineless cactus	Increase in UFA making the lipid profile more desirable	Cardoso et al. (2021)
Protein enriched fermented feed (generated by cardboard box treatment with *Phanerochaete chrysosporium* and *Pleurotus ostreatus* fungi) along with 200 g lucerne hay.	Lower ADG (962 g) and FE (FCR = 7.7) with increasing levels	‐	Decrease in dressing percentage (399 g carcass/kg LW) with increase of protein enriched fermented feed. Bone proportion increased with increase in protein‐enriched fermented feed.	‐	Malik et al. (1999)
Diets containing rumen unprotected betaine (RUPB) (1.6 g/kg) and rumen protected betaine (RPB) (2.9 g/kg)	No effects observed	PUFA content decreased in rumen protected betaine, n‐3 PUFA increased in rumen unprotected betaine. Reduced content of saturated fatty acids, and increased contents of MUFA, and n‐6 PUFA in subcutaneous fat	‐	Decreased water loss (12.42% and 13.63% with RUPB and RPB), shear force (22.55 and 15.54 for RUPB and RPB), increased a* (13.52 with RUPB), but did not affect L* and b*	Jin et al. (2021)
Macadamia oil and vitamin E supplementation to control diet which included Tifton‐85 hay, ground corn, soybean oil, dicalcium phosphate and mineral premix	Reduced dry matter intake (1091.62 g/day) and improved FE (0.19) with macadamia oil and vitamin E	Greater proportions of C18:3 n3, lower levels of arachidonic acid, higher proportions of C18:1	No significant effects observed	Higher L* (41.30), improved flavor and meat tenderness (7.62) with macadamia oil, reduced levels of lipid oxidation of meat with vitamin E. No other significant differences in a*, and b* of muscle, water loss and shear force with macadamia oil and vitamin E	Dias Junior et al. (2022)
Various levels (0, 1, 2, 3 g) of polyherbal mixture (*Solanum xanthocarpum* and *Hedychium spicatum*) along with basal diet (oat straw, ground sorghum, soybean meal, ground corn, wheat bran, corn gluten, bypass fat, calcium carbonate, salt, vitamin and mineral supplement)	Increased dry matter intake (1.071 kg/day with 2 g polyherbal mixture) and daily weight gain (0.247 kg/day with 1 g polyherbal mixture)	‐	No significant effects observed	‐	Orzuna‐Orzuna et al. (2021)
Chitosan (2 g/kg DM)	Greater final body weight (33.1 kg), dry matter intake (0.88 kg/day), and lower RFI (−0.04)	Higher amounts of oleic‐*cis*‐9 acid, LA, linolenic‐*trans*‐6 acid, arachidonic acid, and EPA	Improved dressing percentage (47.5%) and longissimus muscle area (13.9 cm^2^)	No significant effects on meat color and quality	Pereira et al. (2020)
10% Linseed (along with soybean and barley) and 3.89% of marine algae	Lower ADG (234 g/day) and greater slaughter age (99 days) with linseed and marine algae inclusion	Increased ALA, EPA. Decreased DHA, Decreased n‐6/n‐3 ratio	No significant effects observed	Higher lipid oxidation of meat, reduced flavor, and odor	Urrutia et al. (2016)
Fennel (*Foeniculum vulgare*) seed powder (0, 10, 20 g/kg of DM)	Greater daily gain (235 g) and final weight (46.5 kg)	‐	Greater weights of back muscle (4.07 kg), femur muscle (5.96 kg), lean meat (15.43 kg), and warm carcass (21.99 kg)	‐	Shahsavari et al. (2021)
Calcium salts of fatty acids (0%, 4%, 7%, 11%)	Lower ADG (0.06 kg/day) for 11% calcium salts of fatty acids in diet	No significant differences	Lighter hot carcass weight (23.9 kg) with 11% calcium salts of fatty acids in diet	‐	Seabrook et al. (2011)
Rosemary (*Rosmarinus officinalis* L.) distillation residues (200 g) and linseed (10%)	‐	Increased levels of n‐3 PUFA and C18:3n‐3, decreased n‐6/n‐3 ratio with linseed. Rosemary + linseed delayed oxidation of lipids	‐	More tender meat and no significant effects on meat color with Rosemary distillation residues	Ben Abdelmalek et al. (2020)
Rumen bypass fats including prilled fat, prilled fat plus lecithin, calcium salts of fatty acid	‐	Increased UFA and decreased SFA with prilled fat and prilled fat plus lecithin	‐	No effects on cooking loss, drip loss, and shear force. Higher pH with prilled fat plus lecithin (mean around 5.80)	Behan et al. (2021)
Various levels of soybean meal (0%, 33%, 67%, and 100%) replaced by crushed crambe	‐	Linear increase in PUFA (LA and ETA) and no influence on MUFA	‐	Increased flavor and meat tenderness. No effects on the color, shear force and cooking loss	Carneiro et al. (2016)
3% Linseed and 4% of soybean meal	No significant effects on live weight and body weight gain	Higher PUFA, lower n‐6/n‐3 ratio with linseed. Higher levels of PA with soybean	High carcass fatness with linseed (10.71). Less carcass fat with soybean (8.89)	No significant effects on meat pH, and meat color	Facciolongo et al. (2018)
Lupin (358 g DM/day), fish meal (168 g DM/day)	‐	Increase in total n‐6 fatty acids in muscle with lupin addition, Higher long‐chain n‐3 fatty acids content fish meal in diet	Heavier slaughter weights (53.9 kg), hot carcass weight (25.8 kg) with lupin, leaner carcasses with fish meal (65.8 cm)	Tougher meat (high shear force (5.7 kg) with lupin)	Ponnampalam et al. (2002)
Malt distillers' grains (ad libitum, 300 g barley + ad libitum silage, 600 g compound pellet + silage ad libitum)	Lower daily gain (0.65 kg DM/day with 600 g compound pellet + silage ad libitum)	Increased PUFA:SFA ratio of lean tissue	‐	‐	Vipond et al. (1995)
*Nigella sativa* seeds (1%), *R. officinalis* leaves (1%), *N. sativa* + *R. officinalis* (1% + 1%)	‐	No significant effects on intramuscular fat	Greater slaughter weight (34.80 kg) and cold carcass weight (14.10 kg) with *R. officinalis* leaves.	Lower cooking loss (30.44%), drip loss (2.95%), shear force (0.93 kg), L* (32.67), and lipid oxidation of *longissimus dorsi* muscle and greater a* (14.08) with *N. sativa* seeds and *R. officinalis* leaves	Odhaib et al. (2018)
Dietary crude protein levels (8% crude protein (low protein) and 13% crude protein (high protein))	Higher final body weight (56.2 ± 2.00 kg), Average daily feed intake (1.66 ± 0.063 kg/day), ADG (0.264 ± 0.0094 kg/day) n high protein lambs	Greater C18:3n3, C18:2c9t11 C20:0 and C15:0 in high protein lambs.	Greater hot carcass weight (26.22 ± 0.97 kg), drip loss (2.09 ± 0.42%) in high protein lambs	High L* (43.23 ± 0.63), a* (24.61 ± 0.23) and b* (17.10 ± 0.30) of meat in low protein lambs	Wang et al. (2021)
Perilla (*Perilla frutescens* L.) seed (0%, 5%, 10%, 15%)	‐	Increased deposition of intramuscular lipids	No significant effects observed	No significant effects observed	Deng et al. (2018)
Corn stubble (25% (NDF‐25) and 29% (NDF‐29)) and non‐hydrogenated lipids (3% CLA‐60, 3% Safflower oil and 3% linseed oil)	No significant effects on daily gain and FE	Increased MUFA and PUFA with supplemental lipids	No significant effects observed	Improved protein content and meat juiciness	Ramírez‐Bribiesca et al. (2021)
Copper source (10 mg copper/kg DM from copper‐lysine, 20 mg copper/kg DM from copper‐lysine, 10 and 20 mg copper/kg DM from tribasic copper chloride)	No significant effects observed	Increased serum triglycerides and NEFF, decreased total cholesterol	No significant differences in hot carcass weight, dressing percentage and longissimus muscle area, reduction in rib back fat and kidney fat	‐	Cheng et al. (2008)
Spirulina (*Arthrospira platensis*) (2, 4, 8 ppm)	Increased initial and final body weight with 8 ppm (22.05 and 48.46 kg), ADG (290 g/day), dry matter intake (1.82 kg/day)	‐	Increased back fat (6.82 mm), body wall fat (9.39 mm) with 8 ppm spirulina in diets	Increased cooking loss (36.67%) with 8 ppm spirulina in diets	Alghonaim et al. (2022)
Restriction of proteins from 13.3% crude protein to 4.3%	No significant effects on the final body weight, decreased ADG (116 g/day) with restricted protein diet	‐	Decreased back fat thickness (3.19 mm), No significant effects on carcass weight and dressing percentage	Increased shear force (3.67 kg)	Ramírez‐Zamudio et al. (2022)
Doses of L‐arginine (500 mg/kg) and nitroarginine methyl ester hydrochloride (30 mg/kg)	‐	‐	‐	Less tenderness in the *semimebranosus* (41.2 N/cm^2^) with L‐arginine	Cottrell et al. (2015)
*Lactobacillus* (1%, 2% and 3%)	‐	‐	‐	Increased b* (4.19) with 1% *Lactobacillus* followed by 3% *Lactobacillus* (4.01), decreased shear force (28.09) with 1% *Lactobacillus*, and cooking loss (27.11) with 2% *Lactobacillus*	Wang et al. (2022)
Influence of concentrate (3% mineral–vitamin supplement (10.0% calcium, 3.5% phosphorous, 8.0% sodium, 4.4% magnesium, 0.4% sulfur, 0.4% zinc, 0.2 manganese, 0.2% iron), 25% faba bean and 72% barley) or pasture finishing diets	‐	Equal proportions of lipids in lambs fed with concentrate and pasture. Higher proportions of SFA and C18:1 in concentrate fed lambs while pasture‐fed lambs had higher C18:2 and C18:3.	‐	Concentrate fed lambs had lower pH (6.54). Healthy meat in Pasture fed lambs were observed	Hajji et al. (2016)
Levels of sunflower cake (control, 5%, 10%, 15%)	‐	‐	‐	Decreased meat tenderness	Melo et al. (2019)
Cottonseed and its co‐products	‐	Less UFA, CLA, and VA in lambs fed whole cottonseed. Lower n‐3 fatty acids and poor n‐6/n‐3 ratio in lambs fed cotton co‐products.	Cottonseed meal resulted in higher hot carcass weight, dressing percentage and *Longissimus Dorsi* muscle area	Higher pH with cottonseed and its co‐products	Paim et al. (2019)
Dietary probiotic (ad libitum hay and concentrate composed of cornmeal along with 1% probiotic (mixture of *Lactobacillus casei* and *Lactobacillus plantarum* P‐8 in 1:1 ratio))	Increase in the body length (71.50 cm)	Increased intramuscular fat content	‐	Increased pH (6.52), decreased cooking loss (28.79%) and shear force (38.87 N)	Zhang et al. (2022)
Rosemary distillation residues (pellets with different levels of Rosemary residues, wheat bran and soybean meal)	‐	Higher PUFA, n‐6 and n‐3, SFA. UFA levels were unaffected	‐	Reduced lipid oxidation, higher oxymyoglobin content	Yagoubi et al. (2018)
Restriction of milk during fattening period	Poor FE (feed: gain ratio of 3.69), lower ADG from weaning to slaughter (164 g/day)	‐	‐	‐	Santos et al. (2018)
Different vegetable oil supplementation (no oil Calcium soap, 4% sunflower oil calcium soap, 4% camelina oil calcium soap)	‐	Elevated levels of n‐3 fatty acids in camelina oil Ca soap (ALA, EPA, DHA), lowest n‐6/n‐3 ratio	‐	‐	Lup et al. (2018)
0.2% Mannan oligosaccharides	No significant effects on final body weight and ADG	Increased UFA and decreased SFA	‐	‐	Liu et al. (2022)
Yerba mate (*Ilex paraguariensis*) (0%, 1%, 2%, 4%)	No significant effects observed	‐	‐	Lighter (Dall'Olio et al., 2010) and tender meat (2.12% shear force) were observed	Lobo et al. (2021)
Responses to concentrate or alfalfa grazing	No significant differences in body weight or ADG	Greater α‐tocopherol content and CLA on alfalfa grazing but no effects on intramuscular fat content	‐	No significant effects observed	Dervishi et al. (2019)
Lipid supplementation (lucerne (ground and pelleted) with 10% and soybean oil)	Higher DMI with lucerne (134 g/kg)	Lower n‐6/n‐3 fatty acids with lucerne	Lower HCW (13.4 kg) with lucerne and decreased muscle proportion (54.4%) with lucerne and 10% soybean oil	No effects observed	Bessa et al. (2005)
Basal diet (oaten: lucerne chaff) plus fish meal (9% DM) and fish oil (1.5% DM)	No significant effects observed	Increased muscle long chain n‐3 fatty acids	Increased HCW (17.8 kg) with fish meal	‐	Ponnampalam et al. (2001)
Mechanical treatment of whole canola seeds (187 g)	‐	Higher total cholesterol (75.6 mg/dL) and high‐density lipoprotein cholesterol (51.1 mg/dL) at slaughter	Slightly lower dressing percentage (42.5%), and moderate carcass grades with whole canola seeds	‐	Huard et al. (1998)
(1) Whole barley grain (60%) plus alfalfa hay (40%; GB‐AH; the basal diet); (2) CP‐AH; (3) CPD	Low body weight gain (16.3 kg), ADG (195 g/day), FE (7.44 kg DM per kg body weight) with GB‐AH diet	Greater SFA proportions with GB‐AH diet High PUFA to SFA ratio with CP‐AH diet	Increased HCW (21.43 and 20.58 kg), cold carcass weight (20.83 and 19.97 kg) and slaughter weight (44.62 and 43.33 kg) with CP‐AH and CPD	‐	Alshamiry et al. (2023)
Microalgae	Increased DMI (mean around 1.311), ADG (mean of 0.24), lower FCR (mean of 5.67)	Increased CLA, EPA and DHA	‐	Decreased shear force (mean of 35.17), drip loss (mean of 3.65) and increased lightness of meat (mean of 40.58)	Orzuna‐Orzuna et al. (2023)

*Note*: Studies that have not assessed the FE, carcass quality, or meat quality traits are denoted with ‘‐‘. (Lightness (L*), yellowness (b*), redness (a*)).

Abbreviations: ADG, average daily gain; ALA, α‐linolenic acid; CLA, conjugated linoleic acid; CP‐AH, concentrate pelleted diet plus alfalfa hay; CPD, complete pelleted diet; DHA, docosahexaenoic acid; DM, dry matter; DMI, dry matter intake; DPA, docosapentaenoic acid; EPA, eicosapentaenoic acid; ETA, eicosatrienoic acid; FCR, feed conversion ratio; FE, feed efficiency; GB‐AH, barley grain–alfalfa hay; LA, linoleic acid; LW, live weight; MUFA, mono unsaturated fatty acid; NDF, neutral detergent fiber; NEFF, non‐esterified fatty acids; PA, palmitoleic acid; PUFA, polyunsaturated fatty acid; RFI, residual feed intake; SFA, saturated fatty acid; UFA, unsaturated fatty acid; VA, vaccenic acid.

Studies have found that increasing the amount of protein in the diet can effectively improve the ADG and FE in ram lambs (Ebrahimi et al., 2007), and influence the quantity of tissues retained as carcass components (Baracos, 2005). Furthermore, ensuring adequate protein in the diet or increasing protein intake can have a positive impact on lean meat content, as observed by Knapik et al. (2017). These findings strongly suggest that dietary protein intake is a crucial factor in optimizing the growth and development of lambs, which can ultimately lead to improved meat quality. Research has demonstrated that restricting feed intake by 5%–15% can lead to improved FE in cattle (Galyean et al., 1999). However, early feed restriction in Merino lambs showed significant and long‐lasting effects on their liver transcriptomic and plasma metabolic profile, which in turn impacted the overall health and well‐being, as well as their ability to efficiently gain weight during the fattening period. Specifically, these effects altered fatty acid metabolism, protein catabolism, and xenobiotic detoxification, which ultimately lead to decreased FE and slower weight gain (Santos et al., 2018).

Various studies (Table 1) have shown diet supplementation with finger millet and corn straw (Chen et al., 2020), licuri (*Syagrus coronate*) cake (Bagaldo et al., 2019), dried lucerne and soybean oil (Bessa et al., 2005), chromium‐yeast (Domínguez‐Vara et al., 2009), spineless cactus (Cardoso et al., 2021), macadamia oil (Dias Junior et al., 2022), chitosan (Pereira et al., 2020), and fennel (*Foeniculum vulgare*) seed powder (Shahsavari et al., 2021) to increase body weight and ADG despite decreases in dry matter intake, thus leading to increases in FE. This increase in body weight and ADG can be traced back to the differing intestinal absorption rates among different diets, which has the greatest impact on weight gain and plays a critical role in the lambs’ ability to extract nutrients from food, ultimately affecting growth and development. These diets also exhibited excellent antioxidant, antimicrobial, and hepatoprotective properties, and were found to be rich in conjugated linoleic acid, and palmitoleic acid which decrease lipogenesis and fat depositions (Bagaldo et al., 2019; Cardoso et al., 2021; Chen et al., 2020; Dias Junior et al., 2022; Domínguez‐Vara et al., 2009; Pereira et al., 2020; Santos‐Silva et al., 2004; Shahsavari et al., 2021). In contrast, protein enriched fermented feed (Malik et al., 1999), polyherbal mixture diet (containing polysaccharides, flavonoids, and saponins) (Orzuna‐Orzuna et al., 2021), and calcium salts of fatty acids (Seabrook et al., 2011), were observed to reduce animal growth by boosting dry matter intake which resulted in decreased FE (Table 1). The reason for the decrease in FE could be attributed to the reduced breakdown and absorption of nutrients In these dietary options, which tend to be less digestible and degradable, and have a lower nutrient absorption rate, ultimately leading to a decrease in FE. The inclusion of dietary crude protein (Wang et al., 2021), lycopene (Jiang et al., 2015), and fennel seed powder (Shahsavari et al., 2021) in the feeding regime resulted in a marked improvement in both carcass and meat quality traits. According to Wang et al. (2021), when lambs are provided with increased amounts of dietary crude protein, their muscle tissue development is found to be more advanced and superior in comparison to lambs that are not given the same dietary supplements. The addition of lycopene supplements resulted in meat with a lower fat content and a higher ratio of poly‐UFA to SFA, which is the recommended ratio for human health (Jiang et al., 2015). This suggests that these diets play a critical role in enhancing the nutritional value and overall quality of meat, thereby providing a significant benefit to consumers. There were no observed changes in the meat fatty acid (FA) profile when crude protein (barley and canola meal with and without fish meal) (Beauchemin et al., 1995), *Nigella sativa* seeds, and *Rosmarinus officinalis* (Odhaib et al., 2018) leaves were supplemented.

The dressing percentage, hot carcass weight (HCW), cold carcass weight, pH, color, water holding capacity, cooking loss, and shear force are some of the parameters that influence the quality of carcass and meat (Ramírez‐Retamal & Morales, 2014). Studies assessed in this review have reported differences in these quality parameters when fed different diets (Table 1). While addition of calcium soap of palm fatty acid (Behan et al., 2021), cottonseed (Paim et al., 2019), and dietary probiotics (Zhang et al., 2022) to diets resulted in increased pH, lambs fed concentrate diets (3% mineral–vitamin supplement, 25% faba bean, and 72% barley) showed a lower pH (Hajji et al., 2016). These results confirm that diets that are high in energy protect against glycogen‐depleting stressors, resulting in lower pH levels. Studies by Facciolongo et al. (2018) and Deng et al. (2018) reported that dietary supplementations of linseed and perilla (*Perilla frutescens* L.) seeds do not exert any differences in meat pH. A decreased shear force was observed in lambs fed two different forms of betaine (Jin et al., 2021), Rosemary (*R. officinalis* L.) distillation residues and linseed (Ben Abdelmalek et al., 2020), *N. sativa* seeds (Odhaib et al., 2018), *R. officinalis* leaves (Odhaib et al., 2018), sunflower cake (Melo et al., 2019), and yerba mate (*Ilex paraguariensis*) (Lobo et al., 2021). The reduced meat shear force observed could be attributed to the higher intramuscular fat content. This finding implies that incorporating these diets into the sheep's feeding regime could enhance the tenderness of meat since tenderness is inversely proportional to levels of collagen present in intramuscular connective tissues (Purslow, 2014) and thus shear force. Vitamin A (Arnett et al., 2007), perilla seeds (Deng et al., 2018), lupin diet (Ponnampalam et al., 2002), spineless cactus (Cardoso et al., 2021), and protein‐enriched fermented feed (Malik et al., 1999) supplementation enhanced the quality of the carcass with greater carcass marbling scores, increased HCW, and higher contents of intramuscular lipids. These diets had lower levels of neutral detergent fiber, which influenced the reduction in the contents of the gastrointestinal tract due to the diets' quick ruminal degradability. Several other investigated diets such as crushed crambe (Carneiro et al., 2016), lycopene (Jiang et al., 2015), macadamia oil (Dias Junior et al., 2022), sunflower cake (Melo et al., 2019), *N. sativa* seeds and *R. officinalis* leaves (Odhaib et al., 2018), chitosan (Pereira et al., 2020), linseed, and marine algae (Urrutia et al., 2016) were reported to yield satisfactory results for the meat quality parameters such as pH, cooking loss, shear force, color, and water holding capacity (Table 1).

To ensure that consumers are provided with a high‐quality product, it is crucial to consider these various dietary factors when developing an optimal sheep production system. As observed in the studies assessed in this review, the type of feed used and associated dietary supplementation can have a significant impact on the FE, carcass quality, and meat quality, affecting not only feed intake, ADG, carcass conformation, and fat content, but also factors such as meat tenderness, juiciness, flavor, and nutritional value.

### Genes influencing sheep FE, carcass quality, and meat quality

Genes play a key role in influencing FE and production traits, in addition to the dietary influences, as observed in multiple studies in this review, revealing several potential genes and QTL (Table 2). Measurements of FE are based on records of sheep feed intake, which is often challenging to measure, particularly in the case of larger sheep farms and grass‐based systems. Automated feed intake recording tools have partially helped but have not eliminated the many challenges involved in this process with factors such as data entry errors, equipment malfunctions, and the need for regular calibration remaining, highlighting the advantages of applying genomic selection implementation (Deng et al., 2016).

**TABLE 2:** Callipyge (*CLPG*), Calpastatin (*CAST*), and Myostatin (*MSTN*) genes and their physiological connections.

Gene	Sheep breed	Traits analyzed	Physiological connections	Reference
*CLPG*, *CAST*	Prydniprovska meat breed	MQ	Improved MQ	Pomitun et al. (2019)
*CLPG*	Karacabey, Kıvırcık, Ramlıç, Hampshire Down × Merino, Merino, German Black‐Head Mutton × Kıvırcık	CQ, MQ	Improved CQ and MQ	Elmaci et al. (2022)
*CLPG*	Dorset, Suffolk, cross breed, texel cross breed	Growth, CQ, MQ	High DP, larger *longissimus muscle* areas, less carcass fat, shorter carcasses, High SF and CL	Abdulkhaliq et al. (2007)
*CLPG*	Half‐sibling Rambouillet	CQ	Higher Muscle mass	Jackson, Miller, et al. (1997b)
*CLPG*	Half‐sibling Rambouillet	CQ	Higher DP, leg and conformation scores, larger *longissimus muscle* areas	Jackson, Miller, et al. (1997a)
*CLPG*	Half‐sibling Rambouillet	Growth, FE	High FE, Average daily feed intake	Jackson, Green, et al. (1997)
*CLPG*	Polypay dams and Dorset sires	Growth, CQ, MQ	Higher DP, protein and ash percentage, less fat over the Rib eye, larger Rib‐eye area, High SF	Srinivasan (1997)
*CLPG*	Dark face crossed lambs	MQ	Less tender meat and meat color stability	Ma et al. (2020)
*CLPG*	Thalli, Kajli and Lohi	MQ	Higher meat content at hindquarter regions	Shah et al. (2018)
*CLPG*, *CAST*	Lori	MQ	High MQ	Nanekarani and Goodarzi (2014)
*CAST*	Kıvırcık lamb	CQ	Lower live weight, back‐fat thickness, skin + back‐fat thickness	Yilmaz et al. (2014)
*CAST*	New Zealand cross bred	MQ	Less tender meat	Byun et al. (2009)
*CAST*	Purebreed Colored Polish Merino	MQ	Associations with drip loss, intramuscular fat content and meat color	Grochowska et al. (2017)
*CAST*	Awassi rams	Growth, CQ, MQ	High average daily gain, High final body weight, High SF, lower CL, lightness of meat	Jawasreh et al. (2017)
*MSTN*	Texel‐sired half‐sib families	Growth, CQ	More muscle, less fat	Johnson et al. (2005)
*MSTN*	New Zealand Romney	Growth, CQ	High mean leg yield, loin yield and total yield	Hickford et al. (2010)
*MSTN*, *ADRB3*	Kajli	Growth, CQ	Associations with growth traits and carcass traits	Ali et al. (2021)
*MSTN*	Corriedale, NZ Romney, Dorper, Perendale, Merino, Suffolk, Dorset Down, Coopdale, Coopworth, Texel breeds, Poll Dorset, and other cross‐bred sheep	CQ	Improved meat yield	Han et al. (2013)
*MSTN*, *Myogenin*	Baluchi	CQ, MQ	Positive correlations to CQ and MQ traits with improved meat percentage	Forutan et al. (2016)
*MSTN*	Scottish Mule ewes (Blue faced Leicester × Scottish Blackface)	CQ	Lower fat scores and heavier carcasses	Masri (2013)

Abbreviations: CL, cooking loss; CQ, carcass quality; DP, dressing percentage; MQ, meat quality; SF, shear force.

#### Callipyge, calpastatin, and myostatin

Molecular genetics in animal research has facilitated the identification of several genes directly or indirectly associated with FE and production traits (Pomitun et al., 2019) including glis family zinc finger 1, leptin receptor, triadin and retinoic acid receptor (Arce‐Recinos et al., 2021). Callipyge (*CLPG*), *CAST*, and myostatin (*MSTN*) genes (Table 2) are among the most studied with their expression and interaction been shown to affect growth performance, carcass attributes such as carcass weight and meat quality traits including meat tenderness (Arce‐Recinos et al., 2021; Bagatoli et al., 2013).

The *CLPG* gene, located on chromosome 18 was discovered in a flock of Dorset sheep in 1983 (Cockett et al., 1994), and is distinguished by ‘polar overdominance’, a distinct form of nonmendelian inheritance in which the *CLPG* phenotypic expression occurs in a heterozygous population with the mutation paternally inherited (+_Mat_/*CLPG*_Pat_) (Lewis & Redrup, 2005). Numerous studies have shown the *CLPG* gene to be strongly associated with growth and development of muscle tissue and is a valuable DNA marker of increased meat yields (Gorlov et al., 2020). One of the potential advantages of incorporating lambs with a *CLPG* mutation in production systems is its ability to improve profitability (Busboom et al., 1999). *CLPG‐*mutated lambs exhibit muscle hypertrophy leading to abnormal growth of specific thigh muscle groups. Numerous studies (Abdulkhaliq et al., 2007; Elmaci et al., 2022; Jackson, Green, et al., 1997; Jackson, Miller, et al., 1997a, 1997b) (Table 2) have investigated FE, carcass quality, and meat quality traits and the distribution of muscles of sheep harboring *CLPG* genes and revealed that lambs with *CLPG* had higher FE, lower average daily feed intake, higher dressing percentage, higher conformation scores, heavier carcass, larger *longissimus muscle* areas and higher mass of excised muscle in torso and pelvic limb. This combination of muscle hypertrophy, minimal fat deposits, growth characteristics, and carcass and meat qualities make the *CLPG* sheep a unique breed with distinct physical characteristics. Investigations on *CLPG* gene polymorphisms by Gorlov et al. (2020) revealed monomorphic *CLPG* locus with homozygous AA genotype and *CLPG* allelic variants in Russian sheep breeds such as Kalmyk, Volgograd, and Edilbay. Related results were observed in Iranian Lori (Nanekarani & Goodarzi, 2014), Karakachan (Dimitrova & Bozhilova‐Sakova, 2016), and Saudi (Alakilli, 2015) breeds with only the AA genotype. However, *CLPG* studies by Jackson, Green, et al. (1997) and Shah et al. (2018) reported an allelic G mutant in sheep breeds such as Hampshire, Rambouillet, Dorset, and Thalli, resulting in a distinctive appearance of the animal. In terms of the growth, and carcass and meat qualities, sheep with *CLPG* mutation had improved growth (Jawasreh et al., 2019), and the best carcass qualities with good yield of sirloin, whole limbs, shoulders, and loin bones (Busboom et al., 1999).

The *CAST* gene is an inhibitor of calpains and controls the proteolytic activity of enzymes that cause post‐mortem myofibril degradation and tenderness of meat (Bagatoli et al., 2013). Mutations in *CAST* gene that increase *CAST* activity or its production result in a decrease in calpain activity which further results in a reduction in normal skeletal development. Additionally, mutations that increase *CAST* activity or its synthesis also lead to reduced postmortem proteolysis of myofibril proteins which are essential components of muscle tissue, and their breakdown after death is a crucial process that affects the quality of meat (Pomitun et al., 2019). In a previous study on beef steers, *CAST* activity was found to be higher in feed‐efficient animals compared to feed‐inefficient animals, and the meat was more tender (Blank et al., 2017). Similarly, steers selected based on increased residual feed intake (RFI) had 13% increased *CAST* activity when compared to feed‐efficient steers which is then believed to reduce the activity of calpains, leading to a decrease in protein degradation and an increase in net muscle protein accretion (McDonagh et al., 2001). This mechanism may contribute to increased muscle efficiency, as less protein breakdown would result in a greater net muscle gain. The *CAST* gene serves as a marker to evaluate body weight, growth, carcass composition, and meat quality attributes in swine (Koćwin‐Podsiadła et al., 2003) and bovine (Casas et al., 2006). However, only few studies have addressed the subject in sheep so far, and thus taking into consideration critical economic aspects, a relationship between quantitative features characterizing sheep FE, carcass quality, meat quality, and *CAST* polymorphism need to be further explored. According to Pomitun et al. (2019), mutations in the *CAST* gene was found to be polymorphic with two alleles M and N with frequencies 0.83 and 0.17 respectively, and lambs with N allele had an increased live body weight (Table 3). Yilmaz et al. (2014) discovered a mutation in the *CAST* gene that was linked to back‐fat thickness and skin + back‐fat thickness of the loin eye area, as well as ADG. Dorset sheep carrying the ‘ac’ genotype of the *CAST* gene exhibited markedly higher daily live weight gain and produced carcasses weighing 12%–17% more compared to those carrying the ‘aa’ and ‘ab’ genotypes (Palmer et al., 1999). Kumar et al. (2018) studied carcass and meat quality traits in lambs belonging to two *CAST* alleles M and N and observed that sheep with N allele had lower shear force indicating greater meat tenderness and higher ash percentage. The results of all these reported studies have consistently indicated that polymorphisms in the *CAST* gene could play a crucial role in determining traits such as FE, carcass quality, and meat quality. Therefore, it is reasonable to consider the *CAST* gene as a potential candidate for further research in the field of sheep breeding genetics.

**TABLE 3:** Summary of allele frequencies and genotypes of Callipyge (*CLPG*), Calpastatin (*CAST*), and Myostatin (*MSTN*) genes.

Gene	Sheep breed	Alleles (frequencies)	Genotypes (frequencies)	Reference
*CLPG*	Prydniprovska	‐	AA (1.00) (monomorphic)	Pomitun et al. (2019)
*CLPG*	Lori	‐	AA (1.00) (monomorphic)	Nanekarani and Goodarzi (2014)
*CLPG*	Kajli, Lohi, and Thalli	A (0.93), G (0.07) in Thalli breed, A (Abdulkhaliq et al., 2007) in kajli and Lohi breeds.	AG (0.10), AA (0.88), GG (0.02) in Thalli breed. No *CLPG* mutations in Kajli and Lohi breeds.	Shah et al. (2018)
*CAST*	Prydniprovska	M (0.83), N (0.17)	MN (0.13), MM (0.77), NN (0.10)	Pomitun et al. (2019)
*CAST*	Awassi	M (0.486), N (0.514)	MN (0.648), MM (0.352), NN (0.00)	Jawasreh et al. (2017)
*CAST*	Kıvırcık	M (84.24%), N (15.76%)	MM (72.91%), MN (22.66%), NN (4.43%)	Yilmaz et al. (2014)
*CAST*	Lori	A (0.635), B (0.365)	AA (0.32), AB (0.63), BB (0.05)	Nanekarani and Goodarzi (2014)
*MSTN*	New Zealand Romney	A (68.3%), B (22.7%), C (9.0%)	AB (30.2%), AA (46.6%), AC (13.3%), BC (3.5%), BB (5.8%), CC (0.6%)	Hickford et al. (2010)

Similar to *CAST*, the *MSTN* gene which produces the myostatin protein is expressed in skeletal muscles, inhibits proliferation of myoblast and muscle fiber production (Cieślak et al., 2003). A mutation in the *MSTN* gene allows the gene to express less, resulting in increased muscle mass and muscle cell hyperplasia (Bagatoli et al., 2013). *MSTN* has previously been suggested as a possible gene in sheep for increasing muscle production (Han et al., 2013) and its polymorphisms have been reported in various species such as cattle (Casas & Kehrli, 2016), chicken (Zhang, Ran, et al., 2019), and horses (Dall'Olio et al., 2010). In sheep, variations in the *MSTN* gene have already been linked to growth of muscles which are determinants of FE and meat quality attributes (Grochowska et al., 2019). Two single nucleotide polymorphisms (SNPs), c.18 G>T and c.241 C>T, were reported by Osman et al. (2021) with associations to body weight and ADG in Egyptian sheep, suggesting that *MSTN* genes with these SNPs lead to lambs with greater body weight and higher ADG. However, effects of *MSTN* polymorphism on FE related traits may vary depending on sheep breed, with contrasting results reported in several studies (Grochowska et al., 2019). For instance, Ma et al. (2016) and Wang et al. (2016) found that polymorphisms in the *MSTN* gene affected birth weight in Tan sheep and New Zealand Romney breeds, although Kijas et al. (2007) and Johnson et al. (2009) reported no effects on postnatal growth in Australian sheep, Lincoln, Poll Dorset, White Suffolk, or New Zealand Texel breeds. Investigations on the effects of *MSTN* alleles on carcass and meat quality traits have shown that homozygous lambs with *MSTN*‐A/*MSTN*‐A had heavier loin, less fat and decreased meat tenderness (Grochowska et al., 2019) (Table 2). Furthermore, homozygous AA lambs exhibited superior quality carcass with higher dressing percentage, topside and short loin in Australian sheep (Haynes et al., 2013). Han et al. (2013) reported on the variability of the *MSTN* gene and its associations with traits for growth and carcass in New Zealand sheep breeds, finding 28 nucleotide variants (promoter region (*n* = 3), exon 1 (*n* = 1), 5′‐untranslated regions (*n* = 3), intron 1 (*n* = 11), intron 2 (*n* = 5), 3′‐untranslated regions (*n* = 5)). These allelic and genotypic effects of the polymorphisms in the *MSTN* gene reveal its potential to be used as another marker gene in addition to *CLPG* and *CAST* in selecting sheep with better FE, carcass quality, and meat quality. The allele frequencies and genotype frequency of the *CLPG*, *CAST*, and *MSTN* genes in multiple studies have been found to vary slightly, potentially due to the diversity in sheep breeds studied (Table 3).

#### Genes involved in fat deposition in sheep

In addition to the most studied *CLPG*, *CAST*, and *MSTN*, genes and genetic polymorphisms related to fat metabolism (Moradi et al., 2022; Zhang, Gao, et al., 2019) and fat deposition (Dong et al., 2020) have been identified across several studies (Table 4). Because of the complexity of the systems governing fat metabolism, sheep breeding places a high value on controlling fat deposition to increase meat yield, and to improve the quality of meat, breeds with different fat deposition patterns are cross bred (Li et al., 2018). Transcriptomic analysis of two Chinese fat‐tailed sheep breeds by Li et al. (2018) identified four genes: fatty acid binding protein 4 and 5 (*FABP4*, *FABP5*), fatty acid translocase (*CD36*) and adiponectin (*ADIPOQ*) to be abundant in fat tissues. *FABP4* gene has important roles in the transport of FA, deposition of fats and tenderness of meat (Xu et al., 2011). While the *CD36* gene can bind to long chain FA and is crucial in the storage and absorption of dietary lipids (Zhang et al., 2011) the *ADIPOQ* gene which is released by adipocytes regulates glucose and fatty acid oxidation (Tschritter et al., 2003). Polymorphisms in *ADIPOQ* reported to be also related to fat deposition in pigs (Dall'Olio et al., 2009). Additionally, other genes namely, transcription factor 7 (*TCF7*), calcium channel, voltage‐dependent L type, α1F subunit (*CACNA1F*), bone morphogenetic proteins 15 (*BMP15*), solute carrier family 35 member A2 (*SLC35A2*), protein phosphatase 2 catalytic subunit α (*PPP2CA*), and prostaglandin D2 receptor (*PTGDR*) are reported to have roles in fat deposition in sheep (Moradi et al., 2022). Bandi et al. (2019) looked for polymorphisms and domain variations in two genes, myosin binding protein C1 (*MYBPC1*), and septin 7 gene (*CDC10*), respectively, and observed monomorphic and polymorphic patterns with associations to fat storage and carcass marbling. Furthermore, the allele A was found to be more frequent, and the AA genotype had the highest genotypic frequency when compared to BB genotypes. Hence, these allelic forms of *MYBPC1* and *CDC10* could be used in breeding programs to improve marbling and meat quality. Dong et al. (2020) analyzed fat‐tailed (*n* = 18) and thin‐tailed (*n* = 14) breeds genomically and proposed that the platelet derived growth factor D (*PDGFD*) gene could be a target gene for tail‐fat accumulation with *PDGFD* expression showing an association with homeostasis and maturation of adipocytes. Additionally, the same study demonstrated that across species *PDGFD* expression is inversely correlated with adipocyte maturation. The same gene is reported to be highly expressed in many sheep breeds with fat‐tails (Fan, 2015; Liu et al., 2015). Genetic analysis of thin‐tailed breed Zel and fat‐tailed breeds Baluchi and Lori‐Bakhtiari revealed 573 and 242 copy number variations (CNVs), representing 328 and 187 CNV regions (CNVRs) respectively, covering a 73.85‐Mb stretch of the ovine genome with 790 overlapped genes (Taghizadeh et al., 2022). Analysis of the functional enrichment of the identified genes revealed that CNV‐containing genes in thin‐tailed breeds were involved in adaptive immunological response, reactive oxygen species regulation, biosynthesis, and starving response, whilst genes discovered in fat‐tailed breeds were involved in protein cellular modification processes (Taghizadeh et al., 2022). Zhu et al. (2016) also discovered multiple CNVRs (*n* = 738) in different breeds such as Large‐tailed Han (*n* = 371 CNVRs), Tibetan (*n* = 66 CNVRs), and Altay (*n* = 301 CNVRs) sheep, which corresponded to 3130 genes in the ovine genome with involvement in fat deposition and peptide receptor functions. These genes included peroxisome proliferator‐activated receptor‐α (*PPARA*), Kruppel‐like factor 11 (*KLF11*), retinoic X receptor A (*RXRA*), fatty acid synthase (*FASN*), adipocyte determination and differentiation factor 1 (*ADD1*), platelet‐derived growth factor‐α (*PDGFA*), and phosphoprotein phosphatase 1 catalytic subunit A (*PPP1CA*; Table 6).

**TABLE 4:** Genes associated with fat depositions in sheep.

Genes (associated traits)	Breed	Traits analyzed	Reference
*CDC10* and *MYBPC1* (carcass marbling)	Lori‐Bakhtiari and Zel	Marbling	Bandi et al. (2019)
*HGFAC* and *LRPAP1*	Baluchi, Lori‐Bakhtiari, and Zel	Fat deposition	Taghizadeh et al. (2022)
*NPR2* (fat depth), *SPAG8* (carcass weight), *HINT2* (body weight), *CACNB3* (meat tenderness), *PTGDR*, *DDX23* (hot carcass weight, birth weight), *SKP1*, *PPP2CA*, and *TCF7* (fat thickness)	Lori‐Bakhtiari and Zel	Fat deposition	Moradi et al. (2012)
*TCF7*, *PPP2CA* (*OAR5*), *PTGDR*, *NID2* (*OAR7*), *CACNA1F*, *EBP*, *AR*, *HSD17B10*, *BMP15*, *SLC35A2*, *RBM3* (*OAR X*), and *WDR13* (fat metabolism)	Lori‐Bakhtiari and Zel	Fat deposition	Moradi et al. (2022)
*DTNBP1*, *FBF1*, *SMURF2*, *RBM11*, *SETD7* (fat metabolism), *GAB1*, and *SMARCA5* (body size)	Hulun Buir	Fat deposition and body size	Zhang, Gao, et al. (2019)
CNVs identified on *PPARA*, *KLF11*, *RXRA*, *FASN*, *ADD1*, *PDGFA*, *PPP1CA*, and *PEX6* (fat deposition)	Han, Altay, Tibetan sheep	Fat deposition	Zhu et al. (2016)
*HOXC9*	Hu and Tibetan sheep	Fat deposition	Fei et al. (2022)
*PDGFD*	South Asian breeds, European sheep breeds, American sheep breeds, Nepalese sheep breeds, Middle East sheep breeds, African sheep breeds, Chinese sheep breeds	Fat deposition	Dong et al. (2020)

### Heritability and correlations between sheep FE, carcass quality, and meat quality traits

Heritability is a key metric in the field of genetics, as it helps to determine the extent to which genetic differences contribute to the observed variability in a given trait (Turner & Corander, 2021). By contrast, a genetic correlation refers to the proportion of variance shared by two traits (Astles et al., 2006). Table 5 provides a comprehensive overview of the numerous studies that have assessed heritability estimates for FE, carcass quality, and meat quality traits in sheep. The heritability estimates for RFI were observed to ranging from 0.11 to 0.45 (average heritability = 0.28) (Cammack et al., 2005; Johnson et al., 2022; Paganoni et al., 2017; Snowder & Van Vleck, 2003; Tortereau et al., 2020) and for feed conversion ratio (FCR) ranging from 0.1 to 0.3 (average heritability = 0.20) (Snowder & Van Vleck, 2003; Tortereau et al., 2020) suggesting that these traits are moderately heritable (average heritability estimate between 0.20 and 0.30). In addition, the estimates for carcass quality traits were quite varied, with the heritability estimates of HCW ranging from 0.17 to 0.35 (average heritability = 0.28) (Massender et al., 2019; Mortimer et al., 2010, 2014, 2018) and carcass dressing percentage ranging from 0.21 to 0.24 (average heritability = 0.22) (Mortimer et al., 2010, 2018) indicating moderate heritability of these traits. Conversely, the heritability estimates for intramuscular fat were higher, ranging from 0.39 to 0.58 (average heritability = 0.48) (Mortimer et al., 2010, 2014, 2018), indicating that this trait is highly heritable (average heritability estimate >0.30). In case of meat quality traits, heritability estimations were reported for two specific traits in sheep: pH and shear force where heritability values for pH were found to range from 0.1 to 0.15 (average heritability = 0.11) (Mortimer et al., 2010, 2014, 2018) and shear force to range from 0.1 to 0.27 (average heritability = 0.18) (Mortimer et al., 2014, 2018) indicating that these traits are less heritable (average heritability estimate <0.20). The wide range of heritability estimates for multiple traits could be attributed to various factors. Heritability is a population parameter that may vary depending on factors such as the sample size, breeds, management strategies and the diets they are fed. Additionally, variations in the accuracy and length of recording sheep feed intakes, as well as measurements of carcass weights and other meat quality parameters, could also contribute to the variability in heritability estimates.

**TABLE 5:** Heritability estimates for FE, carcass quality, and meat quality traits in sheep.

Trait	Number of sheep/lambs	Breed	Heritability estimates (mean ± SE)	Reference
Feed efficiency				
RFI	1239	Composite rams (½ Columbia, ¼ Hampshire, ¼ Suffolk)	0.11 ± 0.05	Cammack et al. (2005)
RFI	2816	Merino sheep	0.17 ± 0.07	Paganoni et al. (2017)
RFI	1047	Targhee lambs	0.26 ± 0.07	Snowder and Van Vleck (2003)
RFI	986	New Zealand maternal sheep	0.42 ± 0.09	Johnson et al. (2022)
RFI	951	Romane rams	0.45 ± 0.08	Tortereau et al. (2020)
FCR	951	Romane rams	0.3 ± 0.08	Tortereau et al. (2020)
FCR	1047	Targhee lambs	0.1 ± 0.05	Snowder and Van Vleck (2003)
Carcass quality				
HCW	6771	Merino sheep	0.35 ± 0.06	Mortimer et al. (2010)
HCW	16 565	Canadian crossbred lambs	0.17 ± 0.02	Massender et al. (2019)
HCW	8968	Crossbred and Merino sheep	0.25 ± 0.04	Mortimer et al. (2014)
HCW	9135	Merino sheep	0.35 ± 0.1	Mortimer et al. (2018)
IMF	6771	Merino sheep	0.39 ± 0.05	Mortimer et al. (2010)
IMF	8968	Crossbred and Merino sheep	0.48 ± 0.05	Mortimer et al. (2014)
IMF	9135	Merino sheep	0.58 ± 0.11	Mortimer et al. (2018)
DP	9135	Merino sheep	0.21 ± 0.11	Mortimer et al. (2018)
DP	6771	Merino sheep	0.24 ± 0.05	Mortimer et al. (2010)
Meat quality				
pH	4110	Merino sheep	0.1 ± 0.03	Mortimer et al. (2010)
pH	8968	Crossbred and Merino sheep	0.08 ± 0.02	Mortimer et al. (2014)
pH	9135	Merino sheep	0.15 ± 0.07	Mortimer et al. (2018)
SF	9135	Merino sheep	0.1 ± 0.09	Mortimer et al. (2018)
SF	8968	Crossbred and Merino sheep	0.27 ± 0.04	Mortimer et al. (2014)

Abbreviations: DP, dressing percentage; FCR, feed conversion ratio; HCW, hot carcass weight; IMF, intramuscular fat; RFI, residual feed intake; SF, shear force.

The meta‐analysis has been conducted on the reported heritability estimates of FE, carcass quality and meat quality traits in sheep. The results of the meta‐analysis presented in the form of forest plots (Figures S1–S7) showed heritability estimates for RFI (0.27 ± 0.07), FCR (0.19 ± 0.10), HCW (0.26 ± 0.05), intramuscular fat (0.45 ± 0.04), dressing percentage (0.23 ± 0.05), pH (0.09 ± 0.02), and shear force (0.20 ± 0.08). Based on these findings, it is reasonable to suggest that genomic selection for RFI, HCW, intramuscular fat, and dressing percentage is possible in sheep, as these traits have moderate to high heritability estimates (>0.20). However, for meat quality trait (pH and shear force), the heritability estimate from the meta‐analysis was lower (<0.20), indicating the need for further investigations on a larger number of sheep with diverse breeds to validate this information. It is worth noting that the number of studies considered for each cluster of FCR, dressing percentage, and shear force was restricted to only two, as there were very few studies that reported heritability estimates for these traits. As a result, the findings for these traits may not be as conclusive or reliable as those with a larger number of studies.

Genetic correlations can greatly impact the overall quality of the end product such as differences in meat tenderness, flavor, intramuscular fat contents, and cooking loss (Grochowska et al., 2017, 2019, 2021). Table 6 shows the estimated genetic correlations between various FE traits, carcass quality, and meat quality traits which reveals that there are positive genetic correlations between FE traits such as daily feed intake, ADG, RFI, and FCR suggesting that animals with higher feed intake, faster weight gain, and lower RFI tend to have better feed conversion efficiency. However, the studies also reported some conflicting observations. For instance, two studies conducted by Cammack et al. (2005) and Tortereau et al. (2020) found negative genetic correlations between RFI and ADG (−0.03 ± 0.20, −0.03 (0.20)), indicating that animals with higher RFI tend to have lower ADG. Tortereau et al. (2020) also reported a negative genetic correlation between ADG and FCR (−0.77 (0.09)), suggesting that animals with higher ADG tend to have poorer feed conversion efficiency. These conflicting findings imply that there might be trade‐offs between different FE traits, and that improving one trait might come at the expense of another. Studies on genetic correlations between body weight and carcass and meat quality traits have revealed some interesting findings (Table 7). According to these studies, there is a positive correlation between body weight and HCW, dressing percentage, and shear force (0.91 ± 0.06, 0.35 ± 0.15, 0.27 ± 0.24) (Mortimer et al., 2018). In other words, as body weight increases, so do these traits. However, there is a negative genetic correlation between body weight and intramuscular fat (−0.05 ± 0.11) and pH of *Longissimus lumborum* (−0.19 ± 0.17) and *M. semitendinosus* muscles (−0.20 ± 0.15) at 24 h after slaughter. This means that as body weight increases, these traits decrease. HCW was also found to be negatively correlated with the pH of the *Longissimus lumborum* (−0.32 (0.12)) and *M. semitendinosus* muscles (−0.24 ± 0.19) at 24 h after slaughter (Mortimer et al., 2018), as well as between HCW and shear force (−0.09 ± 0.29, −0.06 (0.10)) (Mortimer et al., 2014, 2018). These correlations are important to consider in the meat industry, as they can impact the quality of meat produced. For example, while a higher body weight can lead to a greater yield in terms of HCW and dressing percentage, it may also result in lower intramuscular fat and pH levels, which can negatively affect the taste and texture of the meat. Therefore, it is important to strike a balance between these different traits when breeding and selecting animals for meat production.

**TABLE 6:** Genetic correlations between FE, carcass quality, and meat quality traits in sheep.

Traits	ADFI	ADG	RFI	FCR	BW	HCW	DP	IMF	pH_24_LL	pH_24_ST	SF	Reference
DFI	‐	0.80 ± 0.10	0.61 ± 0.15	‐	‐	‐	‐	‐	‐	‐	‐	Cammack et al. (2005)
ADFI	‐	‐	0.78 (0.08)	0.10 (0.21)	‐	‐	‐	‐	‐	‐	‐	Tortereau et al. (2020)
ADG	‐	‐	−0.03 (0.20)	−0.77 (0.09)	0.49 ± 0.04, 0.27 ± 0.05	‐	‐	‐	‐	‐	‐	Massender et al. (2019) and Tortereau et al. (2020)
RFI	0.87 (0.05)	−0.03 ± 0.20	‐	0.65 (0.12)	‐	‐	‐	‐	‐	‐	‐	Cammack et al. (2005), Paganoni et al. (2017) and Tortereau et al. (2020)
HCW	‐	‐	‐	‐	0.91 ± 0.06	‐	0.15 ± 0.19	0.04 ± 0.15, 0.06 (0.08)	0.19 ± 0.22, −0.32 (0.12)	−0.24 ± 0.19	−0.09 ± 0.29, −0.06 (0.10)	Mortimer et al. (2014, 2018)
DP	‐	‐	‐	‐	0.35 ± 0.15	‐	‐	‐	‐	‐	‐	Mortimer et al. (2018)
IMF	‐	‐	‐	‐	−0.05 ± 0.11	‐	‐	‐	‐	‐	‐	Mortimer et al. (2018)
pH_24_LL	‐	‐	‐	‐	−0.19 ± 0.17	‐	‐	‐	‐	‐	‐	Mortimer et al. (2018)
pH_24_ST	‐	‐	‐	‐	−0.20 ± 0.15	‐	‐	‐	‐	‐	‐	Mortimer et al. (2018)
SF	‐	‐	‐	‐	0.27 ± 0.24	‐	‐	‐	‐	‐	‐	Mortimer et al. (2018)

Abbreviations: ADFI, average daily feed intake; ADG, average daily gain; BW, body weight; DFI, daily feed intake; DP, dressing percentage; FCR, feed conversion ratio; HCW, hot carcass weight; IMF, intramuscular fat; pH24LL, pH of longissimus lumborum after 24 h; pH24ST, pH of *M. semitendinosus* after 24 h; RFI, residual feed intake; SF, shear force.

**TABLE 7:** Markers associated with growth, feed efficiency, carcass quality and meat quality in sheep.

Breed	Biomarker	Traits	References
Chaka sheep Hu sheep, and small‐tailed Han sheep	CNV of phosphatidylinositol glycan anchor biosynthesis class Y (*PIGY*) (Oar_v4.0 36 121 601–36 125 200 bp)	Growth	Feng et al. (2020)
Suffolk, New Zealand Romney	Variations in the promoter regions of uncoupling protein 1 (*UCP1*)	CQ	Yang et al. (2014)
Hu	Rib‐eye associate genes like *LOC105611989*, *DPP6*, and *COL12A1*	CQ	Zhao et al. (2022)
Severokavkazskaya	Polymorphism in *myocyte enhancer gene; factor‐2* (*MEF2B*)	MQ	Trukhachev, Belyaev, et al. (2016)
Small‐Tailed Han sheep, Tan sheep, and Inner Mongolia	Polymorphisms in fast skeletal muscle troponin C (*TNNC2*)	MQ	Xu et al. (2008)
Small‐Tailed Han sheep, Tan sheep, and Inner Mongolia	Polymorphisms in fatty acid binding protein 4 (*FABP4*)	MQ	Xu et al. (2011)
Tattykeel Australian White, Poll Dorset, Texel and Rambouillet	SNPs in stearoyl‐CoA desaturase (*SCD*), fatty acid binding protein‐4 (*FABP4*), and fatty acid synthase (*FASN*)	MQ	Pewan et al. (2021)
Hu sheep	Mutation in adenylyl cyclase 8 (*ADCY8*)	FE	Yang, Wang, et al. (2022)
Hu sheep	Polymorphisms in malic enzyme 1 (*ME1*), carbonic anhydrase 1 (*CA1*)	FE	Zhang et al. (2021)
Ram dual purpose and blackface breed. Dual‐purpose rams (*n* = 205) were primarily composed of Rambouillet and BF rams (*n* = 125) were composed of Suffolk and Hampshire breeds	*GLIS* family zinc finger 1 (*GLIS1*), interleukin 1 receptor accessory protein‐like 1 (*IL1RAPL1*), and SRY sex determining region box‐5 and ‐6 (*SOX5* and *SOX6*)	FE	Cockrum et al. (2012)
New Zealand Romney	Polymorphisms in exon 3 of Insulin‐like growth factor 1 (*IGF1*)	Growth, CQ	Li et al. (2021)
New Zealand Romney	Variations in myostatin (growth and differentiation factor 8 (*GDF8*))	Growth, CQ	Han et al. (2013)
Santa Ines	Polymorphisms in growth hormone and insulin‐like growth factor 1 (*IGF1*)	Growth, CQ	Meira et al. (2019)
Colored Polish Merino	Polymorphism in *IGF1R* c.654G > A (*IGF1*)	Growth, MQ	Grochowska et al. (2021)
Soviet Merino and Salsk sheep breeds	Polymorphism in calpastatin gene (*CAST*)	Growth, MQ	Gorlov et al. (2016)
Santa Ines × Dorper	Enolase 3 (ENO3), malate dehydrogenase (MDH) and retinal dehydrogenase (ALDH1A1))	CQ, MQ	Melo et al. (2019)
Suffolk × Dorset crossbreds and Rideau Arcott	Isopropyl alcohol, aminoadipic acid, acetone for RFI, total dimethylarginine, citric acid, hypoxanthine, hippuric acid, asymmetric dimethylarginine, l‐phenylalanine, and semimembranosus C16:1 for carcass yield grade, lysoPC a C26:1 for carcass muscle‐to‐bone ratio	FE, CQ	Goldansaz et al. (2020)

Abbreviations: CQ, carcass quality; MQ, meat quality; RFI, residual feed intake.

### Other putative omics markers of FE, carcass, and meat quality traits in sheep

Genomic selection methods have opened new opportunities to select animals for breeding purposes with the availability of SNP markers (Meuwissen et al., 2001). The basis for this entails selecting QTL linked with a specific phenotype (Ibtisham et al., 2017) and is advantageous for traits that are less heritable and difficult‐to‐measure. Markers (biological markers or biomarkers) serve as indicators of several biological processes and pathological states, providing valuable insights into various health, production and disease traits. Identification of these biomarkers involves the process of manipulating specific regions that are associated with a desirable trait of interest, which allows for more accurate and efficient selection of desired traits in animals during the breeding process (Ribaut & Hoisington, 1998). Compared to selection based on phenotype such as live weight gain, selection based on markers offers an advantage in that the trait of interest is linked to a molecular marker, which enhances the accuracy and efficiency of selecting the desired trait. This is because phenotype‐based selection relies solely on external characteristics, which can be influenced by environmental factors and may not accurately reflect the genetic makeup of the organism. With markers, the selection process can be targeted towards specific, desirable traits at the molecular level (Jiang, 2013). In recent years, there has been a growing trend of identifying markers on a wide range of genes that are of significant economic importance to better understand their role in various biological processes. However, the understanding of markers in sheep, especially for FE, is still limited. It is crucial to comprehend these markers as they can shed light on the negative impacts of selecting for FE on other important sheep traits such as carcass and meat quality, which can be used to design more balanced breeding programmes (Cantalapiedra‐Hijar et al., 2018).

#### Genetic markers for FE, carcass quality, and meat quality

Based on the findings of the studies examined in this review, polymorphisms in various reported genes can be used as markers in genomic analyses which can be detected using sequencing technologies such as genomics or genome‐wide association studies that enable the generation of massive amounts of genotype data, resulting in a variety of SNP combinations that affect FE, carcass quality, and meat quality traits (Cardona Tobar et al., 2020). Several genes have been identified in this review as having the potential to be employed as markers for economically relevant traits in sheep (Table 7) (Meira et al., 2019; Trukhachev, Belyaev, et al., 2016; Yang et al., 2014). These marker genes include phosphatidylinositol glycan anchor biosynthesis class Y (*PIGY*) with a role in cell‐to‐cell interactions (Feng et al., 2020), uncoupling protein 1 (*UCP1*), which is associated with body composition and variations in growth rate (Yang et al., 2014), myocyte enhancer gene factor‐2 (*MEF2B*), which stimulates myostatin production and further limits growth of muscles in sheep (Trukhachev, Belyaev, et al., 2016), and insulin‐like growth factor 1 (*IGF1*), which is a mediator of growth hormonal effects. A CNVR was identified in the exon 2 of the *PIGY* gene that overlapped with 28 QTL associated with weight of the carcass, muscular density and growth traits such as weight of the body, circumference of chest which are traits with high economic value (Feng et al., 2020). However, the CNV distribution was different among the different breeds of sheep studied (Chaka, Hu, small‐tailed Han) suggesting that this CNV could be breed‐specific. Despite the fact that there was a link between CNV in the *PIGY* gene and economically relevant sheep traits such as growth and carcass quality, there had been no earlier studies on its potential to be used as a marker in sheep or other livestock and Feng et al. (2020) was the first to reveal its capability to be utilized as a marker. Yang et al. (2014) was the first to study variations of *UCP1* gene and its relationship with carcass traits and reported three variants namely variants A, B, and C in the promoter region of *UCP1*. Variant B had lower scores for fat compositions and an increased proportion of lean meat yield, suggesting that genetic selection for this variant of *UCP1* could assist breeders in the future enabling them to pick lambs that are leaner and have better yield. The same gene with two SNPs in the intron region was also identified by Yuan et al. (2012) in sheep in addition to observations in cattle (Chen et al., 2018). *IGF1* gene polymorphisms have been reported consistently in three studies (Grochowska et al., 2021; Li et al., 2021; Meira et al., 2019) in concordance with reports in other species such as cattle (Yang, Zhu, et al., 2022), pigs (Bouquet et al., 2021), and fish (Chandhini et al., 2021) suggesting *IGF1* to be a possible marker of growth and FE. Polymorphisms in the intron region of *IGF1* was found to be associated with carcass traits such as length of carcass and rib yield (Meira et al., 2019). Two other studies report SNPs in the intron 1 region to have associations with sheep body weight (Trukhachev, Skripkin, et al., 2016) and carcass traits (Su et al., 2014). Two *IGF1* genotypes namely A_2_ and B_2_ were detected by Li et al. (2021) where A_2_ was associated with increased HCW and B_2_ with decreased HCW. Furthermore, SNPs in the *MEF2B* and *IGF1* genes had connections with indices of meat quality and were reported to be employed as markers for meat quality in sheep (Trukhachev, Belyaev, et al., 2016; Yang et al., 2014).

#### Protein and metabolite markers for FE, carcass quality, and meat quality

Even though QTL, alleles, and genes have been utilized in several breeding programs, not all identified structural or expression variation at the genetic level necessarily results in the expected phenotype. In this case, proteomics and metabolomics are useful approaches since they focus on the analysis of proteins and metabolites, rather than DNA, which may not always correlate with corresponding downstream protein expression and translation (Weckwerth, 2011a, 2011b). Proteomics and metabolomics are gaining popularity among researchers who want to understand variations in FE and quality of carcass and meat (Zhang et al., 2023). Moreover, integrating genomics with proteomics and metabolomics can discover markers and underlying biological or biochemical mechanisms related with FE, carcass quality, and meat quality attributes without having to know anything about the production or processing conditions that generate differences in FE or meat qualities (Purslow et al., 2021). Table 7 shows the reported protein and metabolite markers for the important production traits in sheep. Melo et al. (2019) assessed changes in the semi‐membranous proteome in lambs when fed sunflower cake and identified changes in three differentially expressed structural proteins (enolase 3, malate dehydrogenase and retinal dehydrogenase) to be correlated with meat tenderness. This was in concordance with findings in other livestock such as beef cattle where the same proteins were correlated with meat quality parameters including juiciness, texture, and tenderness (Gagaoua et al., 2018). Whilst to date only a limited number of studies have found metabolite biomarkers associated with FE and other economic traits, the plasma levels of the hormones thyroxine (*R* = 0.435) and adrenocorticotropic hormone (ACTH; *R* = 0.534) have been reported to be positively correlated with FE (RFI) (Zhang et al., 2017) indicating that low‐RFI or feed‐efficient lambs possess lower concentrations of thyroxine and ACTH. Higher ACTH concentrations can increase excitability and cause a significant loss of energy in the form of heat leading to a decreased FE (Zhang et al., 2017). Paula et al. (2013) assessed the serum levels of metabolites and enzymes and reported that RFI was associated with metabolism of proteins such as albumin (*R* = 0.74) and creatinine (*R* = −0.45). Less feed‐efficient (High‐RFI) lambs had higher albumin levels (3.62 g/dL in High‐RFI lambs and 3.51 g/dL in Low‐RFI lambs), which are associated with levels of feed intake and supply of nutrients, and lower creatinine levels (0.74 mg/dL in High‐RFI lambs and 0.86 mg/dL in Low‐RFI lambs) (Paula et al., 2013). In another metabolomics‐based study, Goldansaz et al. (2020) utilized quantitative techniques to identify several metabolite markers from sheep serum with acetone, isopropyl alcohol, and aminoadipic acid associated with RFI, hypoxanthine, total dimethyl arginine, hippuric acid, citric acid to carcass yield grade, and lysophosphatidylcholine a C26:1 to carcass muscle‐to‐bone ratio.

## CONCLUSIONS AND FUTURE PROSPECTIVES

Considering that feed expenditure accounts for more than 60% of total costs in sheep production systems, selecting animals for FE can help mitigate against this and increase profitability. It is also important to consider additional attributes such as carcass and meat quality, which may be positively or negatively affected by selection of sheep based on FE metrics. This systematic review has examined the current state of knowledge regarding dietary and genetic factors influencing FE and carcass and meat quality and has reported putative genetic, protein, and metabolite markers for these production traits in addition to their heritability estimates and correlations. Incorporating specific diets into the feeding regime of sheep has shown a reduction in dry matter intake and ADG while increasing the final body weight, thereby resulting in enhanced FE. Several diets including vitamin A, finger millet and corn straw, licuri cake, dehydrated lucerne and soybean oil, spineless cactus, macadamia oil, and fennel seed powder were shown to have reported improvements in FE. It is worth noting that the optimal quantity of each diet needs to be carefully considered with moderate levels of some diets found to enhance FE, whereas higher levels of the same diets decreased FE. In other words, determining the optimum amount of each diet is critical to achieving maximum FE. In addition to improving FE, a range of diets incorporating dietary crude protein, chromium‐yeast, spineless cactus, and protein‐enriched fermented feed supplements were shown to improve meat and carcass quality and result in higher HCW, increased carcass fatness, and heavier slaughter weights. Diets containing licuri cake, lycopene, linseed, rosemary distillation residues, and soybean have been found to enhance meat quality, leading to increased color index, flavor, tenderness, and unsaturated fatty acid levels. These findings demonstrate the key role which the incorporation of specified dietary constituents at optimum quantities into feed can play in improving FE whilst maintaining and increasing carcass and meat quality.

The genetic basis of phenotypic variances in important economic traits were also assessed as they are breed characteristics that are crucial and strongly linked to production and welfare aspects. Heritability estimates for FE, carcass quality, and meat quality traits were observed to vary significantly, with some being influenced by genetics (RFI, HCW, intramuscular fat) whilst others were not (pH, shear force). The review also clearly indicates that significant progress has been made in identifying genetic factors that impact production traits in sheep. For instance, a range of genes and genetic polymorphisms, including *CLPG*, *CAST*, and *MSTN*, which are crucial in regulating muscle hypertrophy, growth, live weight, and the quality of carcass and meat have been discovered. Moreover, genes such as *PDGFD*, *PPP2CA*, *PTGDR*, and *HOXC9*, which contribute to tail fat deposition, and *IGF1*, *MEF2B*, *TNNC2*, *FABP4*, and *ADCY8*, which play roles in enhancing FE, carcass quality, and meat quality, have also been identified. It is crucial to pay attention to utilizing these identified genes in sheep breeding programs through marker‐assisted and genomic selection methods. However, further research is necessary to fully understand other novel genes that can impact these traits. Limit attempts have been made to explain the pathways and mechanisms underlying the observed variability in FE, carcass quality, and meat quality in sheep using advanced techniques such as proteomics and metabolomics. Comprehensive investigations applying a systems‐wide biology approach are required to better integrate data from these omics‐based methodologies to develop a broader picture of the physiological basis to these traits. Whilst the applied use of identifiable causal mutations in genes and levels of specific proteins and metabolites as part of a combined marker panel may aid breeding value prediction leading to more consistent selection across breeds and management categories, validation using experimental studies to confirm the potential of these biomarkers are still required.

## CONFLICT OF INTEREST STATEMENT

The authors declare no conflicts of interest.

## Supporting information


Figures S1–S7

## Data Availability

The data that support the findings of this study are available from the corresponding author upon reasonable request. This data is intended for use by researchers and scholars for the purpose of replication, validation, and further exploration of the results and conclusions presented in the manuscript. Requests for data should be directed to the corresponding author for consideration.
