# Supplementary material for: Insights into the influence of diet and genetics on feed efficiency and meat production in sheep
Source: Anim Genet. 2023 Dec 19;55(1):20–46. doi: 10.1111/age.13383 (PMC10952161; doi:10.1111/age.13383)
Supplement: Supplementary file 1 — Figures S1–S7 [file AGE-55-20-s001.docx]

**Insights into the influence of diet and genetics on feed efficiency and meat production in sheep**

Steffimol Rose Chacko Kaitholil^1,2^, Mark H. Mooney^1^, Aurélie Aubry^2^, Faisal Rezwan^3^, Masoud Shirali^1,2, *^

^1^ Institute for Global Food Security, School of Biological Sciences, Queen’s University Belfast, 19 Chlorine Gardens, Belfast, BT9 5DL, UK.

^2^Agri-Food and Biosciences Institute, Large Park, Hillsborough, BT26 6DR, UK.

^3^Department of Computer Science, Aberystwyth University, Penglais, Aberystwyth, SY23 3FL, UK.

^*^ **Corresponding Author:** [Masoud.Shirali@afbini.gov.uk](mailto:Masoud.Shirali@afbini.gov.uk)

**Supplementary figures:**

Figure S1: Forest plot representing heritability estimates for RFI.


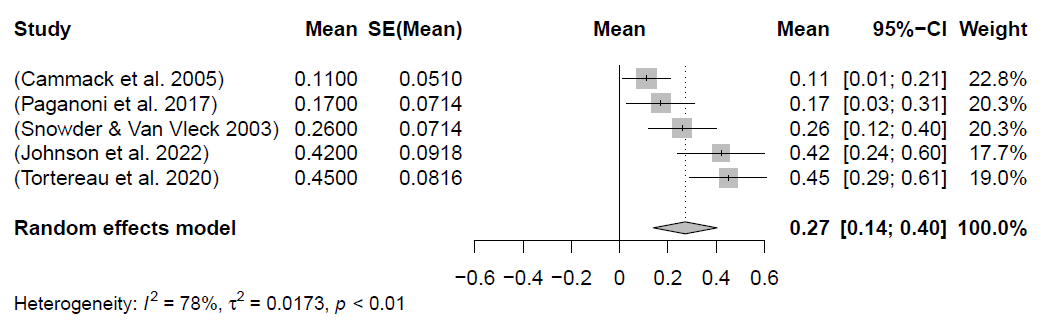


The *I^2^* statistic represents the magnitude of heterogeneity, with 78% showing substantial heterogeneity. τ^2^ or Tau^2^ represents the calculated standard deviation of the underlying effects across the studies. The p-value indicates the overall effect with a p-value < 0.05 indicating a statistically significant difference between the study groups.

Figure S2: Forest plot representing heritability estimates for FCR.


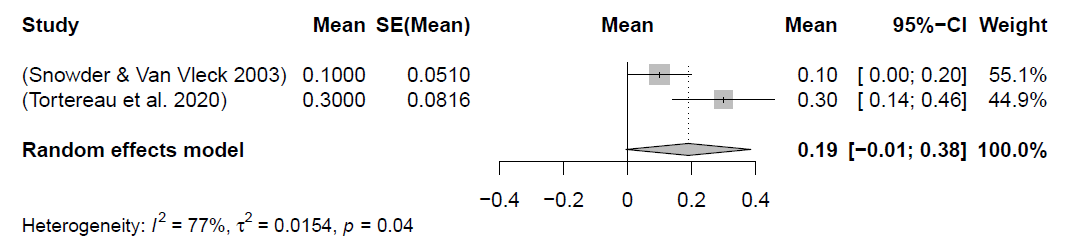


The *I^2^* statistic represents the magnitude of heterogeneity, with 77% showing substantial heterogeneity. τ^2^ or Tau^2^ represents the calculated standard deviation of the underlying effects across the studies. The p-value indicates the overall effect with a p-value < 0.05 indicating a statistically significant difference between the study groups.

Figure S3: Forest plot representing heritability estimates for HCW.


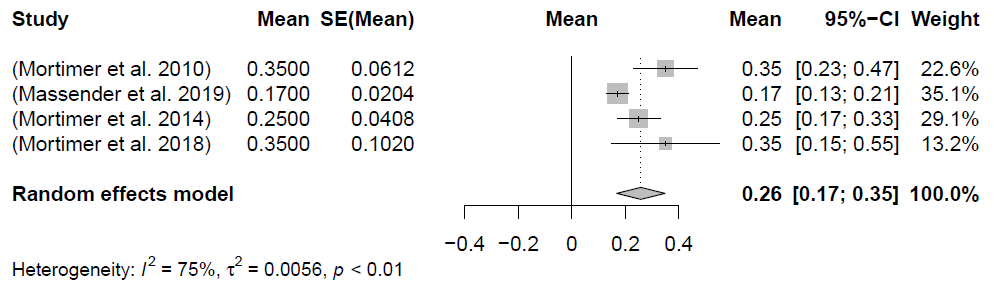


The *I^2^* statistic represents the magnitude of heterogeneity, with 75% showing substantial heterogeneity. τ^2^ or Tau^2^ represents the calculated standard deviation of the underlying effects across the studies. The p-value indicates the overall effect with a p-value < 0.05 indicating a statistically significant difference between the study groups.

Figure S4: Forest plot representing heritability estimates for IMF.


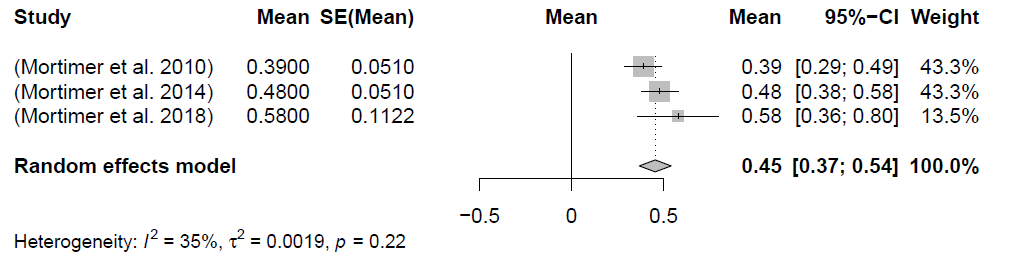


The *I^2^* statistic represents the magnitude of heterogeneity, with 35% showing low levels of heterogeneity. τ^2^ or Tau^2^ represents the calculated standard deviation of the underlying effects across the studies. The p-value indicates the overall effect with a p-value > 0.05 indicating no statistically significant difference between the study groups.

Figure S5: Forest plot representing heritability estimates for dressing percentage.


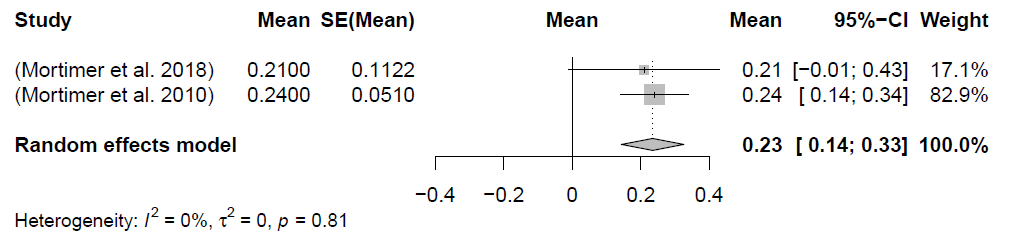


The *I^2^* statistic represents the magnitude of heterogeneity, with 0% showing no heterogeneity observed between the studies. τ^2^ or Tau^2^ represents the calculated standard deviation of the underlying effects across the studies. The p-value indicates the overall effect with a p-value > 0.05 indicating no statistically significant difference between the study groups.

Figure S6: Forest plot representing heritability estimates for pH.


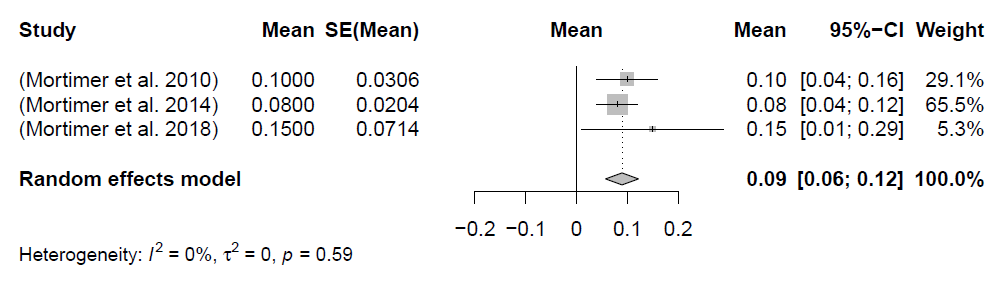


The *I^2^* statistic represents the magnitude of heterogeneity, with 0% showing no heterogeneity observed between the studies. τ^2^ or Tau^2^ represents the calculated standard deviation of the underlying effects across the studies. The p-value indicates the overall effect with a p-value > 0.05 indicating no statistically significant difference between the study groups.

Figure S7: Forest plot representing heritability estimates for shear force.


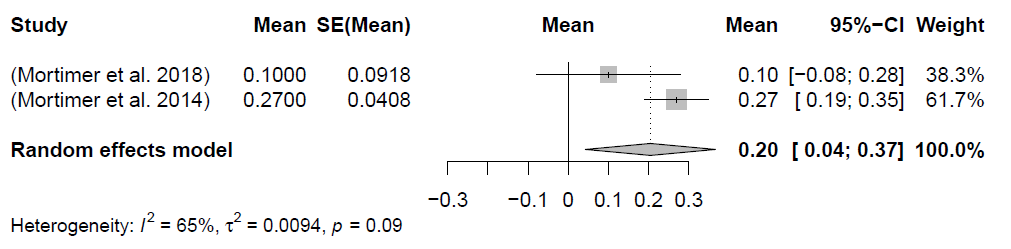


The *I^2^* statistic represents the magnitude of heterogeneity, with 65% showing substantial heterogeneity. τ^2^ or Tau^2^ represents the calculated standard deviation of the underlying effects across the studies. The p-value indicates the overall effect with a p-value > 0.05 indicating no statistically significant difference between the study groups.
